# 

**Published:** 1913-03

**Authors:** 


CONTENTS.

ORIGINAL COMMUNICATIONS:—

Cancer From a Clinical Standpoint, Some Peculiar Observations. By M. B. Hutchins, M. D., Atlanta, Georgia. . . . . . . . . . 599

Some of the Uses of the Laboratory in Diagnosis and Treatment of Disease. By Chas. W. Gould, M. D., Atlanta, Ga. . . . . . . . . . 604

EDITORIAL . . . . . . . . . . 612

SELECTIONS AND ABSTRACTS . . . . . . . . . . 614

BOOK REVIEWS . . . . . . . . . . 644

